# Diaphragmatic breathing for referred pain after total laparoscopic hysterectomy: a randomized clinical trial

**DOI:** 10.1007/s00464-025-12077-6

**Published:** 2025-08-18

**Authors:** Antonio Luis Partida-Márquez, Juan Carlos Fernández-Domínguez, José Antonio Martínez-Fernández, Manuel Pabón-Carrasco, Lidia Melero-Cortes, Ángel Oliva-Pascual-Vaca

**Affiliations:** 1 Red Cross University Nursing Centre, University of Seville, Seville, Spain; 2 Department of Gynecology, Virgen del Rocío University Hospital, Seville, Spain; 3 Institute of Health Sciences Research (IUNICS‐IdISBa), University of Balearic Islands, Carretera de Valldemossa Km 7.5, 07122 Palma, Spain; 4 Department of Nursing and Physiotherapy, University of the Balearic Islands, 07122 Palma, Spain; 5 Madrid School of Osteopathy, Madrid, Spain; 6 Research Group PAIDI-CTS-1050, “Complex Care, Chronicity and Health Outcomes”, Faculty of Nursing, Physiotherapy and Podiatry, University of Seville, 41009 Seville, Spain; 7 Seville Institute of Biomedicine (IBiS), Physiotherapy Department, Faculty of Nursing, Physiotherapy and Podiatry, University of Sevilla, Seville, Spain

**Keywords:** Hysterectomy, Referred pain, Phrenic nerve, Diaphragmatic exercises, Cervical stretching exercises, Laparoscopic surgery

## Abstract

**Background:**

Referred cervicoscapular pain is common after laparoscopic surgery. This pain has different characteristics from incisional pain and requires a different approach.

**Method:**

A blinded, randomized, controlled trial was conducted. Women reporting referred pain with an intensity of 7 points or higher on the visual analogue scale after total laparoscopic hysterectomy (TLH) were randomly assigned to a diaphragmatic breathing group (DBG) or a neck exercise group (NEG). Both groups performed the exercises three times daily, and the usual medical care was maintained. Pain intensity and location were assessed using the McGill questionnaire. Specific self-reported questionnaires were used to assess the evolution of functional disability in the head (HIT-6), shoulder and neck (NDI), or upper limbs (QuickDASH). Follow-up was conducted weekly for 4 weeks after surgery.

**Results:**

Seventy-four women (43.7 ± 9,5 years; 26.6 ± 4.9 body mass index) were recruited. The most common area of referred pain was the shoulder and neck (*n* = 55), followed by the head (*n* = 48) and upper limbs (*n* = 14), with four women showing pain in all three areas and thirty-five in two areas. Both groups showed improvement over time in pain intensity and functional disability in all the locations (*p* < 0,001). However, DBG demonstrated a faster recovery for pain intensity, NDI, and HIT-6 (*p* < 0,001), while no between-group difference was found for QuickDASH (*p* > 0,05). No adverse effects were reported for any of the tested interventions.

**Conclusions:**

Referred pain in TLH tends to become self-limiting over time, but diaphragmatic breathing exercises resulted in a faster reduction in pain intensity and craniocervical disability when compared with gentle cervical stretching exercises. Thus, abdominodiaphragmatic breathing might be a simple and safe complementary intervention to be taught to patients suffering from referred pain after TLH.

Laparoscopic, or minimally invasive, techniques are diagnostic and therapeutic procedures performed at the abdominal and pelvic level that are less harmful than laparotomies, and are associated with a lower degree of pain at the surgical site, fewer days of hospitalization, better aesthetic outcomes, and less blood loss [1–4]. However, laparoscopic procedures are associated with a longer surgical time [2, 5]. Furthermore, in 80% of cases, post-laparoscopy pain occurs in the shoulder, neck, head and/or upper limbs—pain that does not typically occur following laparotomy [1, 3, 6–14].

In addition to the visceral and/or incisional pain perceived at the surgical site [15, 16], laparoscopic procedures require distension of the anatomical cavity using CO_2_ (pneumoperitoneum) to improve visualization by camera [1, 17]. This distension irritates the peritoneum, which is believed to be responsible for the development of referred pain in the cervicoscapular region [1, 3, 9–11], due to its innervation by the phrenic nerve [18, 19]. This type of referred pain does not occur in the postoperative period following laparotomy.

Incisional pain and referred pain are not mutually exclusive, and they exhibit distinct clinical courses and characteristics. Referred pain reaches its maximum intensity 24 h after surgery, shows a poorer response to conventional analgesic treatments, and persists longer than incisional or visceral pain [5, 9]. The occurrence of post-laparoscopic referred pain increases in patients over the age of 50 and is also associated with longer surgical durations [5].

To manage post-laparoscopic referred pain, various procedures have been used [20]. On the one hand, attempts have been made to address it using strategies targeting the presumed cause of the pain, namely subdiaphragmatic irritation due to gas. Thus, the reduction of residual gas, either through aspiration or drainage, has generally yielded positive results [21–23]. In this sense, it has been hypothesized that the positive outcomes observed with Trendelenburg positioning are due to the facilitation of residual gas displacement from the diaphragm to the pelvis, thereby promoting reabsorption [20, 24]. Similarly, with the aim of mobilizing and expelling gas from the abdominal cavity, pulmonary recruitment techniques have been applied. These consist of performing air insufflations prior to extubation and have shown favorable results [11]. To minimize diaphragmatic stretching caused by gas, low-pressure pneumoperitoneum techniques have also been employed, with fairly high levels of evidence supporting their effectiveness in reducing shoulder pain [25]. Beyond causal treatments, the evidence for analgesic and anesthetic interventions is mixed. These include lidocaine patches [26], intraperitoneal administration of anesthetics or dexamethasone [27, 28], ultrasound-guided phrenic nerve block [17, 29, 30], or neuromodulation using TENS [27].

On the other hand, abdominodiaphragmatic breathing exercises involve both analgesic and mechanical mechanisms underlying some of the previously described treatments for post-laparoscopic referred pain, and have been shown to be of interest in patients with neck pain unrelated to laparoscopy [7, 31, 32]. Therefore, the objective of this study is to analyze the effectiveness of self-administered diaphragmatic breathing exercises for the treatment of referred pain in women undergoing total laparoscopic hysterectomy (TLH), either conventional or robotic [33].

## Material and method

### Design

A blinded randomized controlled clinical trial was conducted between February 2023 and June 2024, registered on ClinicalTrials.gov (NCT05959785). The study received approval from the Research Ethics Committee (REC) of the Virgen Macarena and Virgen del Rocío University Hospitals (Seville, Spain), and was carried out in accordance with the ethical standards of the Declaration of Helsinki [34]. All participants were informed about the objectives and procedures of the study, and provided written informed consent prior to participation.

### Participants

The sample was recruited using consecutive sampling of women aged between 18 and 65 years, scheduled for conventional or robotic TLH at Virgen del Rocío University Hospital and Quirón Sagrado Corazón in Seville (Spain). To be included, participants had to present post-laparoscopic pain in the head, neck, shoulder, or arm, with an intensity of 7 or higher on the McGill Visual Analogue Pain Scale within 24 h of surgery. Patients with neurological disorders, cognitive impairment, psychiatric illness, or non-compliance with the assigned therapeutic plan were excluded.

### Randomization and blinding

Simple randomization (1:1) of the participants to experimental interventions was performed using a random number table generated in Excel software (Microsoft, WA, United States of America). Sealed opaque envelopes were used to conceal the treatment allocation. The randomization sequence was maintained by a research assistant who was not directly involved in the trial.

The statistician responsible for the data analysis remained blinded to group assignment and study objectives. Participants were informed that two different types of interventions were being implemented and that they would be randomly assigned to one of them.

### Descriptive variables

Age, body mass index, and parity were recorded. Clinical data were collected on the medical diagnosis that indicated the surgery, history of abdominal or pelvic surgery, type of surgery, duration of surgery, and length of stay from admission to postoperative discharge.

The Brief Pain Questionnaire (BPQ) in its abbreviated version and adapted to the Spanish population [35] was used to determine the analgesic effect attributed to the prescribed pharmacological analgesia and the need to use rescue drugs.

### Outcome variables

The primary outcome variable was the subjective assessment of pain referred to the head, neck, shoulder, or arm. It was measured using the McGill questionnaire in its validated version adapted to the Spanish population [36]. This is a multidimensional pain questionnaire consisting of 66 items in 4 dimensions: current pain intensity in 5 categories (mild/moderate/strong/strenuous/unbearable), pain assessment using a visual analogue scale, pain localization using a body map in which patients can identify the areas where they perceive the painful sensation by using human silhouettes, and subjective qualities of pain [36]. Of the 4 dimensions, pain assessment using a visual analogue scale and referred pain territories were recorded.

In addition, based on the identification of the location of referred pain after laparoscopy, several self-reported questionnaires, validated and cross-culturally adapted to the Spanish language, were used. Specifically, these were the QuickDASH questionnaire for shoulder and/or upper limb pain, the HIT-6 scale for headaches, and the Neck Disability Index (NDI) questionnaire for neck pain.

The QuickDASH questionnaire is an instrument consisting of 11 items answered on a 5-point Likert-type scale and containing questions about symptoms and the ability to use the instruments of the upper limbs during the last week**.** It presents evidence of validity and reliability and has been validated for use in Spanish in its abbreviated version [37].

The HIT-6 scale is an instrument that assesses the negative effects of headache on normal activity using 6 questions/items: Headache frequency, limitation of daily activities (both occupational and social), fatigue, irritability, difficulty concentrating, and response to treatment. Each question is scored between 6 and 13 points, for a total score between 36 and 78 points. Higher scores indicate greater impact, and scores less than or equal to 49 points are considered to have little or no impact. This questionnaire provides evidence of validity and reliability [38] and has been cross-culturally adapted for use in Spanish [39].

The NDI assesses neck pain and its impact on basic activities of daily living. It uses 10 items to measure self-reported disability related to neck pain, and it has shown to be valid when compared with other measures of pain and disability. Each item is measured on a scale of 0 (no disability) to 5 (maximum disability), and an overall score out of 100 can be obtained, which is calculated by summing the scores for each item and multiplying it by two. It provides evidence of validity and reliability and has been cross-culturally adapted for use in Spanish [40].

### Study protocol

The preoperative evaluation was conducted in person one week before the intervention for all women scheduled for surgery during the study period. Data were collected on age, body mass index (BMI), parity, surgical indication, history of abdominal or pelvic surgery, and preoperative medication use. In addition, pain intensity and location were recorded if any preoperative pain was reported in the head, neck, shoulders, or arms.

Total laparoscopic hysterectomy (TLH), either conventional or robotic, was performed under general anesthesia in the Trendelenburg position with a tilt of 15° to 30°. In robotic procedures, the same surgical protocol was followed as in conventional TLH, with the assistance of the da Vinci® robotic system. In both approaches, pneumoperitoneum was established by insufflating CO₂ into the abdominal cavity at a pressure of 14 mmHg. Continuous bladder drainage was required in both surgical approaches.

Regarding the postoperative evaluation, the first assessment was conducted in person 24 h after surgery (T0). It was performed on all patients and consisted of determining the presence of post-laparoscopic referred pain to register its location and intensity. The Brief Pain Questionnaire (BPS) was used to assess the analgesic effect attributed to pharmacological treatment and the need for rescue medication.

Those patients who met the eligibility criteria and agreed to participate, based on the locations of the reported pain, completed the corresponding questionnaires (Quick- DASH, HIT-6, NDI).

Participants were then randomly assigned to one of the experimental interventions of the trial. The assigned intervention was explained in detail, and understanding was verified. Thereafter, a series of post-surgical follow-up assessments were scheduled via telephone to record the outcome variables: week 1 (T1), week 2 (T2), week 3 (T3), and week 4 (T4).

## Interventions

### Diaphragmatic breathing group (DBG)

**Fig. 1:**
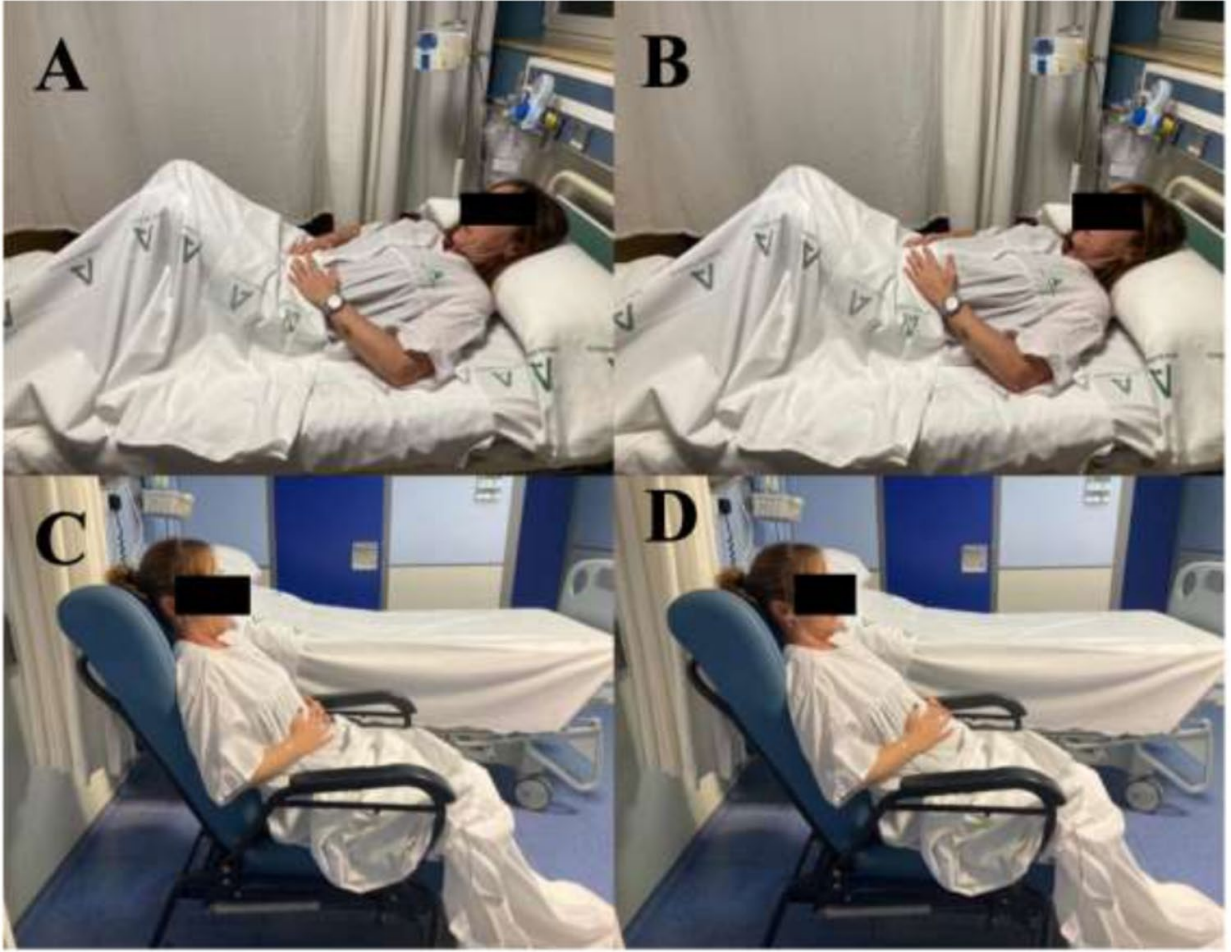
Breathing exercises in sitting and lying position. **A** exhalation in supine position, **B** inhalation in supine position, **C** exhalation in sitting position, **D** inhalation in sitting position

The intervention consisted of self-administered, painless, active abdominodiaphragmatic breathing exercises performed for 5 minutes, three times a day. Although patients could choose the most convenient time, it was recommended that the exercises be carried out before main meals. These exercises began within the first 24 h postoperatively, once the patient had been assigned to this group. Patients were instructed in performing the abdominal breathing exercises both in the supine position and while sitting, and with the hands on the abdomen to assure the diaphragmatic excursion (Fig. 1).

### Neck exercise group (NEG)

**Fig. 2:**
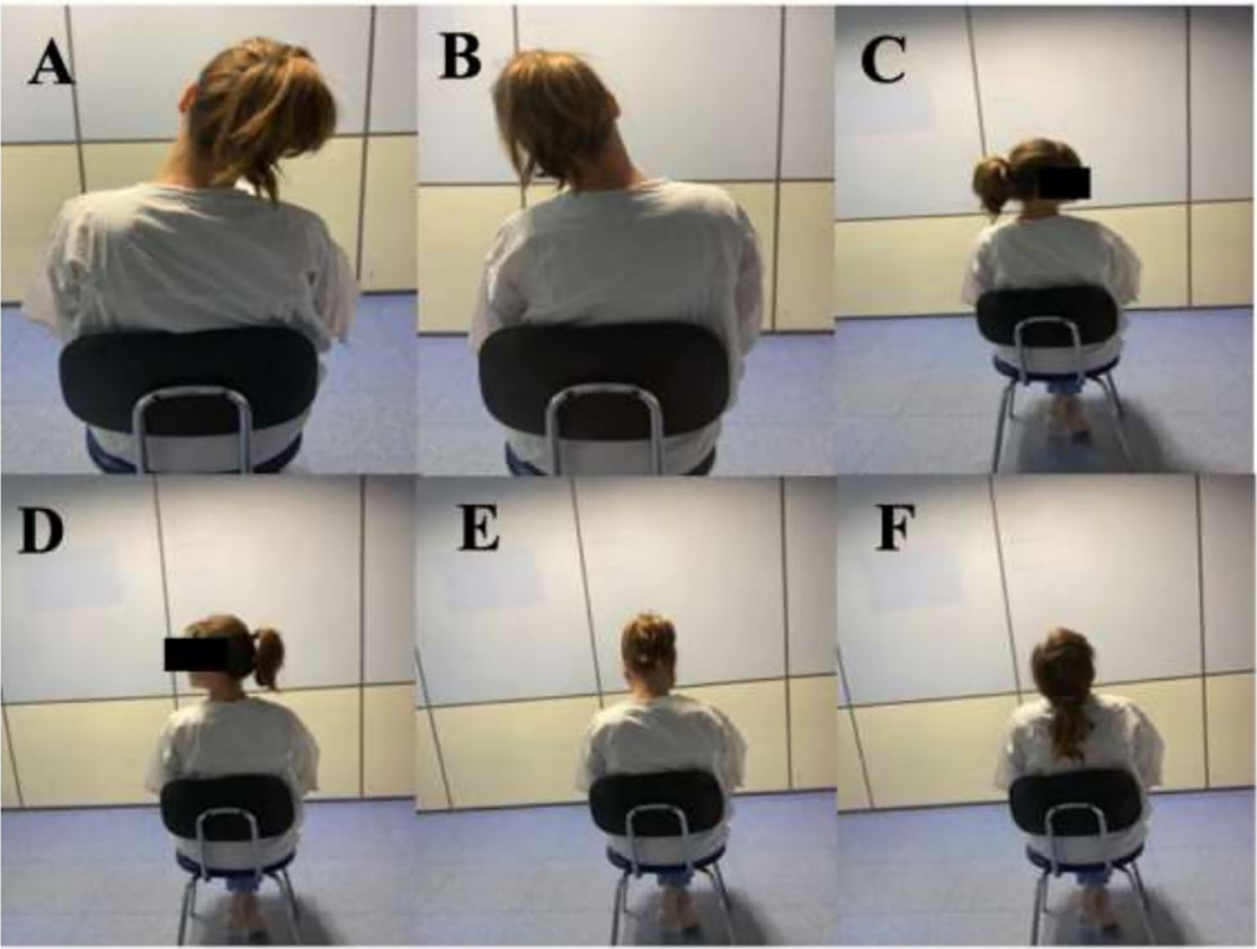
Cervical stretching exercises. **A** Right lateral flexion, **B** Left neck flexion, **C** Right neck rotation, **D** Left neck rotation, **E** Ventral neck flexion, **F** Dorsal neck extension

They performed a self-administered cervical mobilization program of gentle to moderate intensity. Transitions between positions were performed slowly, holding the achieved range of motion for 5 s. This range of motion had to avoid causing or increasing pain. The exercises were also carried out for five minutes, three times a day, and commenced 24 h after surgery (Fig. 2).

At the time of group allocation, healthcare staff explained the exercises in person, answered any questions on-site, and provided a telephone number and email address for support during the follow-up period.

In addition to the exercise programs, all patients received the standard pharmacological analgesic regimen established for this type of surgical intervention. Paracetamol was used as the first-line analgesic (maximum dose: 3000 mg/day), and dexketoprofen as the second-line analgesic (maximum dose: 75 mg/day). These drugs were administered intravenously until oral intake was resumed. In patients with allergies to either drug, alternatives with similar analgesic effects were provided [41–44].

### Statistical analysis

An initial descriptive analysis was conducted for both qualitative and quantitative variables. Quantitative variables were expressed as mean and standard deviation (SD) when normally distributed, or as median and interquartile range (IQR) otherwise. Qualitative variables were presented as absolute frequencies and percentages.

Baseline comparisons between groups were performed using the Student’s *t*-test or the Mann–Whitney U test, depending on normality, and the chi-square test or Fisher’s exact test for qualitative variables.

Postoperative pain progression was assessed using repeated measures ANOVA, including within-group comparisons across five time points and between-group comparisons. Post hoc comparisons were conducted with Bonferroni correction. The Shapiro–Wilk test was used to assess normality, and the Mauchly test for sphericity; Greenhouse–Geisser or Huynh–Feldt corrections were applied as appropriate when sphericity was violated. The effect size was calculated using eta squared (η^2^).

When assumptions for parametric tests were not met, non-parametric equivalents were used. Within-group changes were analyzed using the Friedman test, followed by Wilcoxon signed-rank tests with Bonferroni correction if significant. Between-group comparisons at each time point employed the Mann–Whitney U test.

All statistical analyses were carried out using SPSS version 29.0®, following an intention-to-treat approach. Statistical significance was set at *p* < 0.05.

Accepting an alpha risk of 0.05 and a beta risk of 0.2 in a two-tailed comparison, a minimum sample size of 36 subjects in each group was estimated to detect a difference equal to or greater than 1.7 points in the main outcome variable (subjective pain assessment) assuming a standard deviation of 2.3 points. A 20% loss to follow-up rate was estimated. The sample size was calculated using Granmo v7.12 software (Hospital del Mar, IMIM, Barcelona, Spain) taking into account the results obtained by previous post-surgical studies for the same type of referred cranio-cervico-brachial pain [45, 46].

## Results

**Fig. 3:**
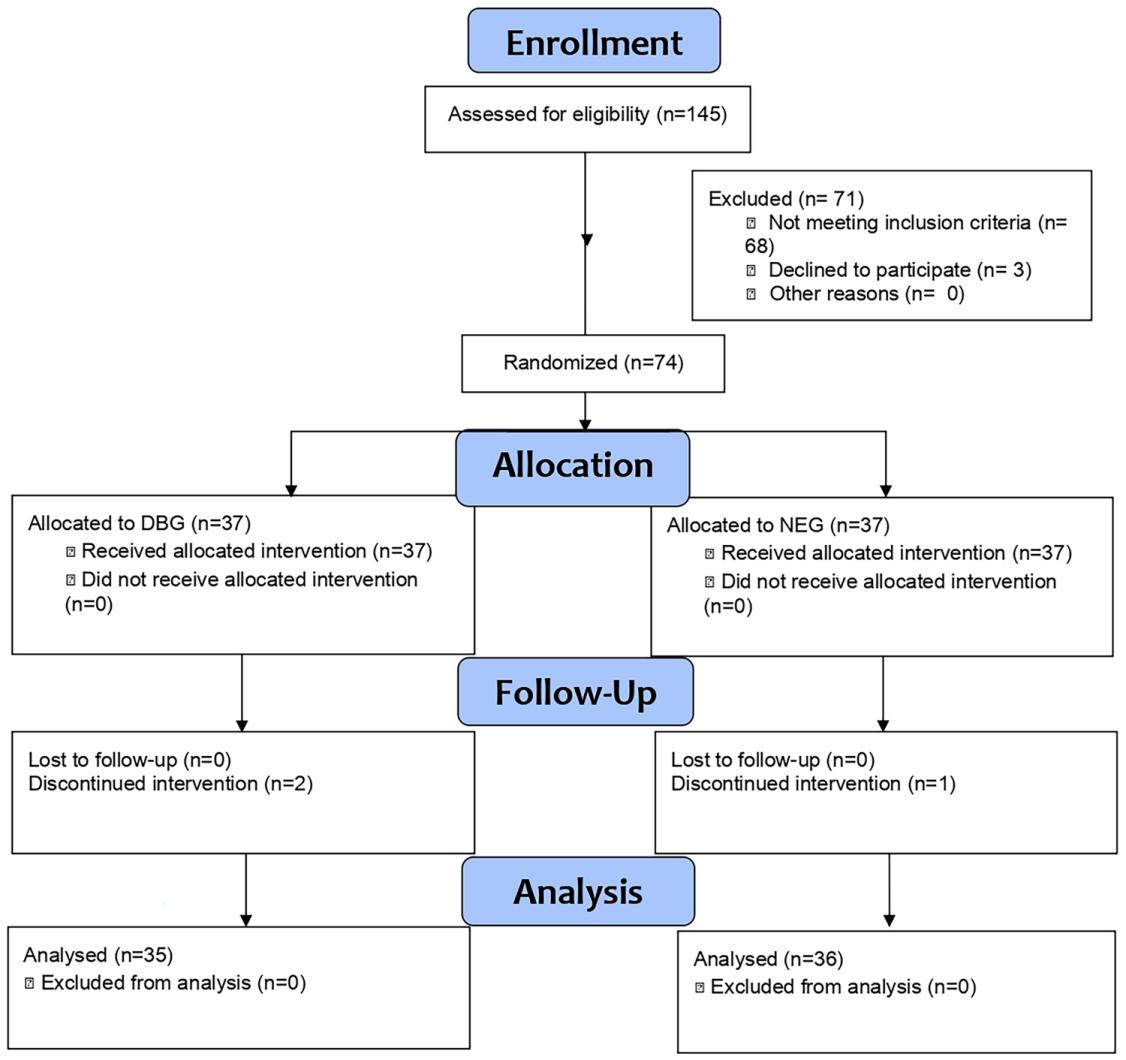
Flowchart of progress through the phases of a two-group parallel randomized clinical trial (i.e., recruitment, intervention assignment, follow-up, and analysis)

A total of 145 women were evaluated. Of these, 77 reported pain with characteristics consistent with postoperative pain following TLH and of sufficient intensity to meet the selection criteria. Three women declined to participate in the study. The remaining 74 participants were randomly assigned to one of the two study groups (DBG or CEG) in similar proportions. During follow-up, two women from the DBG and one from the CEG withdrew from the study due to unwillingness to continue treatment. Thus, 35 women completed the process in the DBG and 36 in the CEG (Fig. 3), as they confirmed during the telephone follow-up that they were adhering to the protocols as explained.

Regarding the locations of the reported pain, four patients experienced pain in the head, neck, and arm; 35 experienced pain in two of these locations; and in 32 patients, pain was limited to one of these areas. Thus, a total of 49 participants experienced pain in the head, 55 in the neck–shoulder area, and 14 in the arm. Arm pain was always concomitant with pain in another location.

Table 1 compares the main sociodemographic and clinical characteristics of the subjects in both study groups.

**Table 1:** Demographic and clinical characteristics of the sample groups

		DBG (*n* = 37)	NEG (*n* = 37)	*P* value
Age in years		52.55 ± 9.12	53.8 ± 8.61	0.987
BMI		25.17 ± 4.86	28.59 ± 4.42	0.006
Parity (number of children)	Childless	5	3	0.076
	1 child	12	11	
	2 children	4	9	
	3 or more children	16	14	
Surgical time (minutes)		286.07 ± 79.17	296.38 ± 86.34	0.976
Medical diagnosis	Oncological	24 (64.9%)	19 (51.3%)	0.155
	Hyperplasia	2 (5.4%)	4 (10.8%)	
	Endometriosis	1 (2.7%)	0 (0.0%)	
	Prophylactic	2 (5.4%)	0 (0.0%)	
	Myomas	7 (18.9%)	10 (27%)	
	Other benign tumors	1 (2.7%)	2 (5.4%)	
	Chronic pain	0 (0.0%)	1 (2.7%)	
	Others	0 (0.0%)	1(2.7%)	
Type of surgery	Robotized	11 (29.7%)	9 (24.3%)	0.54
	Not robotic	26 (70.3%)	28 (75.7%)	
History of abdominal surgery or pelvic	Without prior surgery 1 previous surgery 2 previous surgeries 3 or more previous surgeries	31 (83.8%) 4 (10.8%) 1 (2.7%) 1(2.7%)	28 (75.7%) 6 (16.2%) 3 (8.1%) 0 (0%)	0.372
Days of hospitalization	Days	2.41 ± 1.04	2.38 ± 1.09	0.457
Relief with drugs		4.73 ± 1.48 (4.23–5.22)	4.51 ± 1.50 (4.01–5.01)	0.268

*DBG* Diaphragmatic breathing group. *NEG* Neck exercise group. *BMI* Body mass index

Prior to surgery, the DBG group had a pain intensity in the studied areas of 1.03 ± 2.49, while the NEG group had a pain intensity of 1.35 ± 2.48 (*p* = 0.463). Table 2 presents the pain intensity values and the different questionnaires after surgery, prior to the start of the different exercise protocols (T0).

**Table 2:** Values and between-groups comparison at T0

		DBG	NEG	*P* value
Pain intensity Post-surgery, T0	Mean ± SD	7.62 ± 0.64 (7.41–7.83) *N* = 37	7.68 ± 0.47 (7.52–7.83) *N* = 37	0.340
NDI T0 *n* = 55	Mean ± SD	58.58 ± 8.73 (55.05–62.11) *N* = 27	58.46 ± 2.75 (55.90–61.02) *N* = 28	0.478
HIT-6 T0 *n* = 48	Mean ± SD	63.38 ± 5.15 (61.20–65.55) *N* = 24	62.71 ± 2.75 (61.54–63.87) *N* = 24	0.290
Dash T0 *n* = 14	Mean ± SD	63.28 ± 10.37 (53.69–72.87) *N* = 7	63.28 ± 7.45 (56.39–70.18) *N* = 7	0.948

*DBG* Diaphragmatic breathing group. *NEG* Neck exercise group. *NDI* Neck disability index. This scale assesses neck pain and its impact on basic activities of daily living. *HIT-6* Scale is an instrument that assesses the negative effects of headache on normal activity. *Dash*  Is an instrument about symptoms and the ability to use the instruments of the upper limbs

Table 3 presents the evolution of pain intensity and scores on the various questionnaires throughout the clinical trial. Both groups showed improvement in pain intensity and scores on the various questionnaires over time. However, a statistically significant faster improvement (*p* < 0.001) was observed in favor of the DBG on all of the outcome variables but QuickDASH (*p* > 0.05), with medium effect size for pain intensity (η^2^ = 0.382) and large effects for NDI (η^2^ = 0.748) and HIT-6 (η^2^ = 0.799). Participants in neither study group reported adverse events during follow-up from T0 to T4.

**Table 3:** Inter-group temporal evolution of pain values using the McGill questionnaire (scale 0–10) as well as the NDI value for shoulder and neck, HIT-6 for headache and DASH for the upper limb in the study groups

	Time	Mean, SD, 95% CI		*P* value	
Pain intensity (*n* = 74)		DBG	NEG	Time	Time inter-group
	T0	7.62 ± 0.64 (7.41–7.83)	7.68 ± 0.47 (7.52–7.83)	< 0.001	< 0.001
	T1	5.35 ± 1.49 (4.85- 5.85)	7.11 ± 0.97 (6.79–7.43)		
	T2	2.95 ± 1.41 (2.47–3.42)	5.54 ± 1.41 (5.07–6.01)		
	T3	1.65 ± 0.98 (1.32- 1.97)	3.35 ± 1.55 (2.83- 3.87)		
	T4	1.08 ± 0.83 (0.80–1.36)	1.95 ± 1.15 (1.56–2.33)		
NDI (*n* = 55)	T0	58.58 ± 8.73 (55.05–62.11)	58.46 ± 2.75 (55.90–61.02)	< 0.001	< 0.001
	T1	36.08 ± 14.35 (30.28–41.87)	51.77 ± 8.24 (48.44–55.10)		
	T2	17.54 ± 6.48 (14.92–20.16)	33.31 ± 16.07 (26.81–39.80)		
	T3	5.88 ± 4.64 (3.70–7.45)	10.62 ± 11.40 (6.01–15.22)		
	T4	1.50 ± 1.67 (0.82–2.18)	2.54 ± 4.14 (0.87–4.21)		
HIT- 6 (*n* = 48)	T0	63.38 ± 5.15 (61.20–65.55)	62.71 ± 2.75 (61.54–63.87)	< 0.001	< 0.001
	T1	46.71 ± 5.77 (43.73–48.61)	54.42 ± 8.97 (50.63–58.21)		
	T2	20.83 ± 10.18 (16.53–25.13)	35.96 ± 14.03 (30.03–41.89)		
	T3	7.08 ± 5.65 (4.69–9.47)	14.67 ± 13.85 (8.82–20.52)		
	T4	1.63 ± 1.74 (0.89–2.36)	3.25 ± 5.20 (1.05–5.45)		
DASH^$^ (*n* = 14)	T0	63.28 ± 10.37 (53.69–72.87)	63.28 ± 7.45 (56.39–70.18)		
	T1	42.83 ± 16.86 (27.23–58.42)	49.75 ± 13.78 (37.00–62.50)	< 0.05^$^	> 0.05^$^
	T2	26.40 ± 13.99(13.45–39.34)	28.65 ± 12.64 (16.96–40.35)		
	T3	11.75 ± 2.19 (9.72–13.78)	14.90 ± 7.77 (7.71–22.08)		
	T4	3.61 ± 4.54 (0.59–7.81)	7.74 ± 5.09 (2.73–12.15)		

*DBG* Diaphragmatic breathing group. *NEG* Neck exercise group. *NDI* Neck disability index. *HIT-6* Headache impact test. *Dash* Quick scale. *T0* 24 h post-surgery. *T1* 1 week post-surgery. *T2* 2 weeks post-surgery. *T3* 3 weeks post-surgery. *T4* 4 weeks post-surgery. *$* Mann Whitney U test

## Discussion

The objective of our study was to evaluate the effectiveness of diaphragmatic breathing exercises for the treatment of referred pain in TLH. Our results show that these exercises are more effective than active cervical mobilizations under similar self-administration and dosage conditions in terms of daily repetitions and time spent. Thus, breathing exercises achieve an earlier reduction in referred pain intensity, promoting a faster recovery of function in neck pain and headaches. This faster recovery of function could not be demonstrated for arm pain, where the small sample size (*n* = 14) may have been a factor preventing it, although a better trend in progression was observed in the DBG. Furthermore, since arm pain was always accompanied by pain in either the head or neck–shoulder region, and these areas improved with breathing exercises, it seems plausible that diaphragmatic exercises may also be beneficial for arm pain.

Various strategies are commonly used to address post-laparoscopic referred pain. Some interventions aim to prevent its onset during the surgical procedure. The literature has reported the effectiveness of approaches such as phrenic nerve anesthetic infiltration [17], intraperitoneal irrigation with local anesthetics [28, 47], lung recruitment or forced air insufflations at the end of surgery [11, 48], low-pressure pneumoperitoneum [49], or the insertion of postoperative catheters to eliminate pneumoperitoneum [11]. Additionally, the preventive use of non-steroidal anti-inflammatory patches [50] and perioperative administration of pregabalin [51] have shown promising results. Once referred pain has developed, standard analgesics are used—typically non-steroidal anti-inflammatory drugs [52, 53], with opioids employed as rescue medication [54].

Combinations of two or more pain management strategies (e.g., peripheral nerve blocks, intraperitoneal local anesthetics, systemic analgesics, or local anesthetics with adjuvants) have been shown to offer greater efficacy and longer duration of effect in reducing postoperative pain and opioid use than monotherapies [55]. In fact, opioids are now recommended as part of multimodal therapy or restricted to cases where multimodal analgesia without opioids proves insufficient [56]. According to our findings, diaphragmatic breathing could be added as a complementary strategy, as none of the participants reported adverse effects suggesting harm or interaction with the standard analgesic regimen.

Our results may be explained by the modulation of phrenic nociceptive afferents achieved through diaphragmatic breathing. Nociceptive modulation can occur through several mechanisms, including electrical stimulation [57], mechanical stimulation via external agents such as massage or manual therapy, or—as in our study—through movement, in this case of the diaphragm [58]

On the other hand, according to the Gate Control Theory, the afferences from tissues innervated by the same metameres as the source of pain also have a modulating effect on the painful sensation [59]. In this way, performing cervical exercises such as those developed by the NEG involves a stimulation of the cervical tissues that should contribute to modulating the pain, since these mobilizations also involve the C3–C5 tissues, which correspond to the phrenic metameres. Therefore, our results could indicate that the afferences coming from the affected tissue itself have a greater modulating effect on pain than those coming from other tissues, even if they are from the same metamere.

Another plausible explanation is that diaphragmatic exercises may act directly on the cause of pain, beyond simple neuromodulation. These exercises could facilitate the dispersion of CO₂ retained from pneumoperitoneum, which irritates phrenic nerve endings.

If the cause of the referred pain was irritation of the brachial plexus as a result of the surgical position [60], we could expect a greater analgesic effect from performing cervical exercises, something that has not occurred. In any case, performing breathing exercises can be beneficial since deep breathing and diaphragmatic breathing have an analgesic effect by themselves both in somatic and visceral pain [61–63]. The analgesic effect of deep breathing has been proposed to be due to various factors including cognitive, emotional, and autonomic issues [64].

Regarding the clinical course of pain, the results of our study coincide with those of other authors in that referred pain begins to be perceptible around the first 24 h after surgery [9, 10]. Regarding its duration, the literature indicates that this pain tends to be self-limiting [6], lasting the time it takes for the pneumoperitoneum to resolve [65]. Thus, although there are studies that indicate that it is limited to the first 7 days after surgery [10], other studies indicate that the pain can extend up to 5 weeks [20], as occurred in our study.

Concerning the incidence of referred pain post-laparoscopy, reported rates vary widely between 35 and 80% [7, 12, 66, 67]. For instance, Lee et al. (2018) [10] reported an 80% incidence, but with a mean pain intensity of 4.4. In our study, 51% of patients reported pain of at least 7 points. Therefore, had we included patients with lower pain intensity, the overall prevalence in our study would have been higher. This difference in baseline pain intensity could also explain the longer duration observed in our study compared to Lee’s.

Regarding the study’s limitations, it should be noted that our sample consisted exclusively of women undergoing TLH, limiting the generalizability of our findings to other laparoscopic procedures or male patients. Additionally, we did not systematically assess the specific characteristics of the pain. However, patients who described their symptoms typically referred to the pain as dull or heavy; none used descriptors commonly associated with neuropathic pain, such as burning or tingling. Moreover, although participants were asked about adverse events during follow-up, no specific tool was used to assess satisfaction or adverse effects. Nevertheless, no participant in either group reported noticeable improvement or worsening immediately after performing the exercises. Furthermore, the small number of patients with arm pain warrants caution in interpreting results from the QuickDASH questionnaire. Lastly, women in the NEG group had a higher body mass index, which may also have influenced the results.

## Conclusion

Diaphragmatic breathing exercises can be incorporated into the range of pain management strategies for women experiencing referred pain following TLH. They have been shown to be an effective intervention for achieving a faster reduction in pain intensity, improving cervical function, and reducing headache severity. However, they do not appear to improve upper limb function more rapidly than gentle cervical mobilization.
