# Correction to: Diaphragmatic breathing for referred pain after total laparoscopic hysterectomy: a randomized clinical trial

**DOI:** 10.1007/s00464-025-12156-8

**Published:** 2025-08-28

**Authors:** Antonio Luis Partida-Márquez, Juan Carlos Fernández-Domínguez, José Antonio Martínez-Fernández, Manuel Pabón-Carrasco, Lidia Melero-Cortes, Ángel Oliva-Pascual-Vaca

**Affiliations:** 1 Red Cross University Nursing Centre, University of Seville, Seville, Spain; 2 Department of Gynecology, Virgen del Rocío University Hospital, Seville, Spain; 3 Institute of Health Sciences Research (IUNICS‐IdISBa), University of Balearic Islands, Carretera de Valldemossa Km 7.5, 07122 Palma, Spain; 4 Department of Nursing and Physiotherapy, University of the Balearic Islands, 07122 Palma, Spain; 5 Madrid School of Osteopathy, Madrid, Spain; 6 Research Group PAIDI-CTS-1050, “Complex Care, Chronicity and Health Outcomes”, Faculty of Nursing, Physiotherapy and Podiatry, University of Seville, 41009 Seville, Spain; 7 Seville Institute of Biomedicine (IBiS), Physiotherapy Department, Faculty of Nursing, Physiotherapy and Podiatry, University of Sevilla, Seville, Spain

## Correction to: Surgical Endoscopy 10.1007/s00464-025-12077-6

The original online version of this article was revised to correct affiliation 7. The full affiliation is:

Seville Institute of Biomedicine (IBiS), Physiotherapy Department, Faculty of Nursing, Physiotherapy and Podiatry, University of Sevilla, Seville, Spain.

The original article has been corrected.

